# Effect of large mechanical stress on the magnetic properties of embedded Fe nanoparticles

**DOI:** 10.3762/bjnano.2.31

**Published:** 2011-06-01

**Authors:** Srinivasa Saranu, Sören Selve, Ute Kaiser, Luyang Han, Ulf Wiedwald, Paul Ziemann, Ulrich Herr

**Affiliations:** 1 Institute for Micro- and Nanomaterials, Ulm University, Germany; 2 Institute for Electron Microscopy,Ulm University, Germany,; 3 Institute of Solid State Physics, Ulm University, Germany

**Keywords:** hydrogen in metals, magnetic anisotropy, magnetic data storage, magneto-elastic interactions, nanoparticles, superparamagnetism, thin films

## Abstract

Magnetic nanoparticles are promising candidates for next generation high density magnetic data storage devices. Data storage requires precise control of the magnetic properties of materials, in which the magnetic anisotropy plays a dominant role. Since the total magneto-crystalline anisotropy energy scales with the particle volume, the storage density in media composed of individual nanoparticles is limited by the onset of superparamagnetism. One solution to overcome this limitation is the use of materials with extremely large magneto-crystalline anisotropy. In this article, we follow an alternative approach by using magneto-elastic interactions to tailor the total effective magnetic anisotropy of the nanoparticles. By applying large biaxial stress to nanoparticles embedded in a non-magnetic film, it is demonstrated that a significant modification of the magnetic properties can be achieved. The stress is applied to the nanoparticles through expansion of the substrate during hydrogen loading. Experimental evidence for stress induced magnetic effects is presented based on temperature-dependent magnetization curves of superparamagnetic Fe particles. The results show the potential of the approach for adjusting the magnetic properties of nanoparticles, which is essential for application in future data storage media.

## Introduction

Magnetic data storage has been an integral part of computer system technology for many decades and this will most probably remain so in the near future. Over the years, the basic technology of the hard disc, which allows access to magnetic information stored as individual data bits in a magnetic thin film, has been improved without changing the basic concept of moving a read/write head over a rotating disk surface. The impressive advancement in storage density (usually measured in bits per square inch of disc surface) has been achieved by successive reductions of the bit size leading to higher total capacities. This development is associated with the introduction of new technologies, such as the magneto-resistive read heads first based on the anisotropic magneto-resistance (AMR) and later on the giant magneto-resistance (GMR) and tunnel magneto-resistance (TMR) effect. In conventional magnetic thin films, each bit comprises a large number of magnetic grains, which are coupled by dipolar interactions and, to some extent, by inter granular exchange coupling. The necessity for incorporation of many grains in each bit arises from the requirement for a sufficiently large signal-to-noise ratio. A further increase of the storage density would require a reduction of the size of individual grains.

The magnetization behavior of small particles has been a topic of interest for many years [1–2]. For particle diameters less than a critical size, a single domain state is expected. In such a case, the magnetization of the particle can be represented by one single magnetic moment which adjusts its direction under the influence of local anisotropies, such as the magneto-crystalline anisotropy field, and external fields. In addition, thermal fluctuations may lead to instability of the magnetization over time, as described by Néel [2] and Brown [3]. If the total anisotropy energy *K*_eff_·*V* per grain approaches a lower limit of *K*_eff_·*V* ≈ 50–60 *k*_B_*T*, the magnetization will switch in an uncontrolled way within a period of 10 years which is generally considered as not acceptable for data storage applications; here, *K*_eff_ is the effective anisotropy energy density, *V* is the volume of the particle and *k*_B_*T* the thermal energy. The loss of stability can, in principle, be avoided by the use of materials with high coercivity [4], such as chemically ordered FePt or CoPt alloys. However, the use of such materials is limited by the achievable magnetic field of the write head. Patterned media have been discussed as another possible solution, where the data bits are stored in single grains arranged in a regular manner. This would reduce the noise contribution from the irregular domain (bit) boundaries. Spontaneous self-organization of magnetic nanoparticles, as demonstrated first by Sun and co-workers [5] and subsequently by applying micellar preparation techniques [6], has opened up new possibilities for generating this type of media. Another approach for stabilization of the magnetization in small particles is the coupling to an antiferromagnet [7–8]. This leads to an increase in the coercivity and additionally, to an exchange bias field, which may shift the magnetization curves along the field axis. This approach is similar to the method used for the pinning of the magnetization of the reference layer in spin valve sensors. In this context, it should be noted that magnetic nanoparticles also have applications in other fields, such as medical treatment, diagnostics and imaging [9].

A precise control of the magnetic anisotropy energy is most important for the design of future magnetic data storage media. The total effective magnetic anisotropy *K*_eff_ is a superposition of contributions from magneto-crystalline (*K*_mc_), shape (*K*_shape_), interface (*K*_int_ ) and magneto-elastic (*K*_me_) energies:

[1]
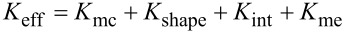


The values of *K*_mc_ range from typically 10^4^ to several times 10^5^ J/m^3^ for Fe, Ni and Co, and up to 6·10^6^ J/m^3^ for FePt at ambient temperature [4]. The maximum of *K*_shape_ is given by 1/2·μ_0_·*M*_s_^2^, which can also reach values around 10^6^ J/m^3^ for typical saturation magnetization *M*_s_ values of about 10^6^ A/m. Since the contribution from *K*_int_ depends on the density of interfaces, it reaches comparable values only when the layer thickness or multilayer periodicities are set in the range of 1 nm. The contribution of *K*_me_ may be estimated for isotropic materials by *K*_me_ = 3/2·λ·σ, where λ is the magnetostriction constant and σ is the mechanical stress. The value of λ varies from typical values of 10^−4^–10^−5^ for most materials up to 10^−3^ for some rare earth alloys. To be comparable with the other contributions to the effective anisotropy, stresses in the GPa (10^9^ Pa) range would be required. However, in thin films and other nanostructured materials plastic deformation by dislocation glide is constrained by the presence of surfaces and interfaces. Therefore, large elastic stresses may be present in these materials. In such systems, *K*_me_ may contribute significantly to *K*_eff_. It has recently been demonstrated that thin CoFe and Ni films subjected to large biaxial stresses show variations of *K*_eff_ of up to 50% [10]. Even larger modifications up to 100% have been achieved in Co/Pd multilayers with perpendicular anisotropy [11]. In these materials, which are interesting as potential perpendicular recording media, the interface anisotropy energy may exceed the shape anisotropy for short multilayer periodicities and cause the magnetization to align perpendicular to the plane of the film in the absence of an external field. The modification of the magnetic anisotropy by large lattice distortions and the generation of additional perpendicular anisotropies has also been demonstrated in the case of CoFe alloys, which show large increases in magnetic anisotropy when subjected to tetragonal distortions by incorporation of the material into CoFe/Pt superlattices [12] or growth on Pd(001) surfaces [13].

In the present contribution, we extend the investigations of the stress effect on the magnetic anisotropy to the study of Fe nanoparticles embedded in a nonmagnetic film. If the particles are spherical and do not experience a strong dipolar interaction, the value of *K*_shape_ should be very low. Bulk Fe has a *K*_mc_ of only about 5·10^4^ J/m^3^ , so that contributions from *K*_me_ should modify the magnetic behaviour of the nanoparticles even at moderate stress values. We first introduce the experimental method for applying stress and provide evidence for the presence of large biaxial stresses. Then we present results from SQUID magnetometry of Fe nanoparticles in the stressed and stress-free state for the same sample over a range of temperatures.

## Results and Discussion

### Deposition and structure of the Fe nanoparticles

**Figure 1:**
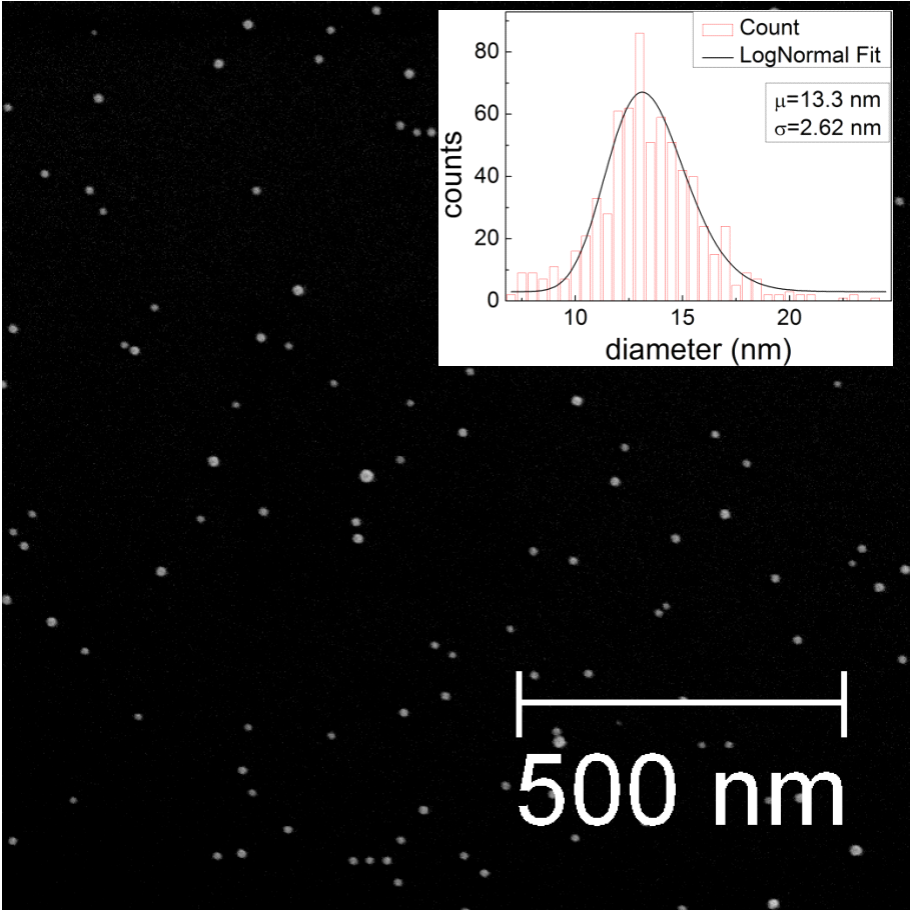
Scanning electron micrograph of Fe nanoparticles deposited on Si. The average particle size observed is 13.3 nm.

**Figure 2:**
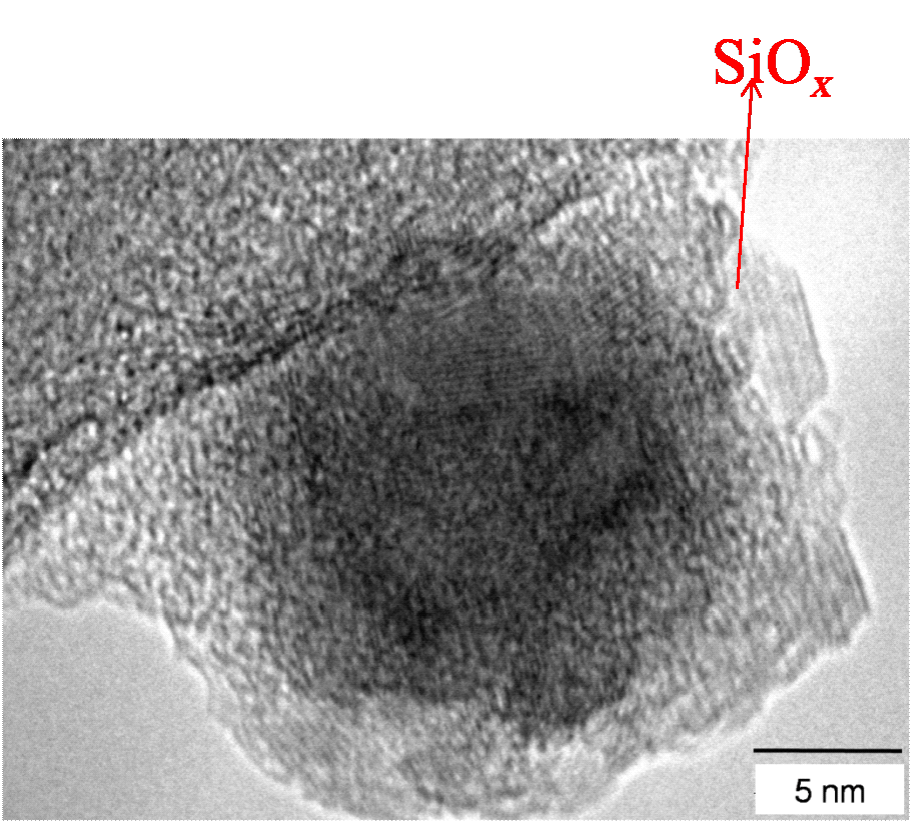
Transmission electron microscope image of Fe nanoparticles (dark contrast) coated with a thin SiO*_x_* layer (brighter contrast).

**Figure 3:**
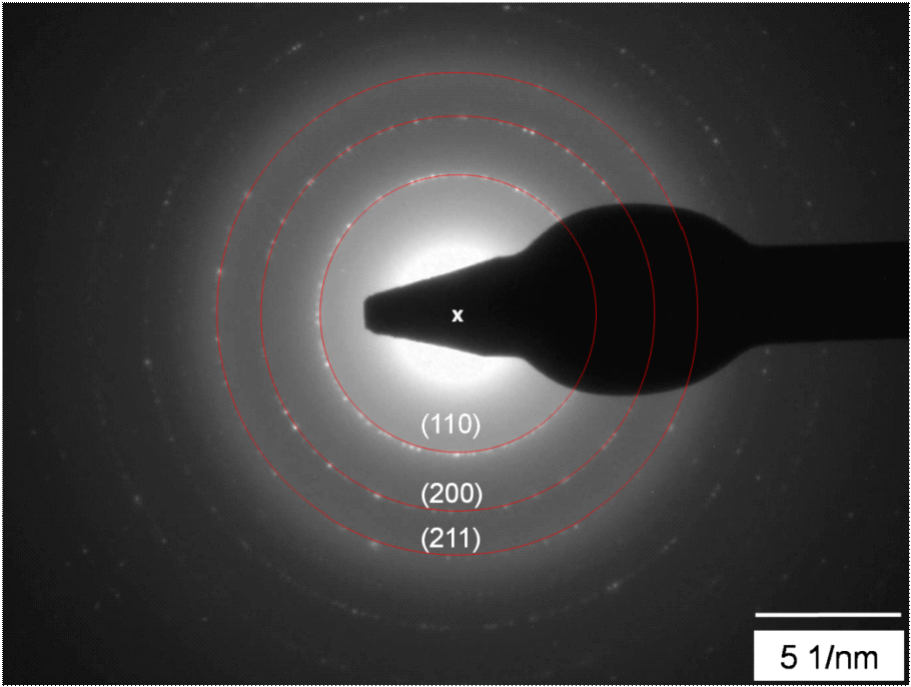
Electron diffraction pattern of the Fe nanoparticles. The Miller indices of the respective lattice planes belong to the bcc structure of Fe.

The Fe nanoparticles used in this study were prepared by plasma-assisted gas phase condensation [14]. This method allows deposition of both, elemental [15] and alloy clusters with rather narrow size distributions. By combination with a film deposition technique, in situ embedding of the clusters is possible. This can not only be used to protect the clusters from oxidation, but also to yield new functionalities such as those exhibited in granular giant magneto-resistance (GMR) systems [16–17] or the introduction of exchange bias effects in nanoparticle systems [18]. Although the arrangement of the deposited clusters on the substrate surface is generally random, it has been recently demonstrated that a self-assembly of the clusters is possible by deposition on a polymer film which subsequently coats and separates the particles [19]. The particle size distribution generated under these conditions was examined by scanning electron microscopy (SEM) and atomic force microscopy (AFM). Figure 1 shows a representative sample of Fe nanoparticles deposited on a silicon wafer. The particle diameters follow a log-normal distribution, typical for the gas condensation technique [20], with a mean size of 13.3 nm and distribution width σ = 2.6 nm. For structural investigations by transmission electron microscopy (TEM), the particles were covered in situ with a thin SiO*_x_* layer to avoid oxidation during the transfer to the TEM. Figure 2 shows a representative TEM image of Fe particles. Different sets of lattice planes can be observed in the particle, proving that the particles themselves consist of sub-particles with different lattice orientations. This indicates that the particles are most likely formed by agglomeration of small primary clusters during transport in the inert carrier gas. Figure 3 shows the electron diffraction pattern obtained from a number of such particles. The superposition of the diffraction spots leads to Debye–Scherrer rings which can all be attributed to Bragg reflections from bcc Fe, proving that the Fe nanoparticles crystallize in the bcc phase.

### Embedding and stress application procedure

In this work, stress was applied to the nanoparticles by increasing the volume of the substrate on which they have been deposited through loading it with hydrogen after the deposition process. This method has previously been successfully applied in the case of thin films of Ni, FeCo and Co/Pd [10–11,21]. Ta foils (thickness 200 μm) were used as substrates, which can be loaded with hydrogen up to concentrations of several 10% at quite low temperatures if a Pd coating is applied to the surface of the Ta [22]. This leads to changes of the linear dimensions of up to 3%. The actual stress generated in the films depends on the transfer of the elastic strain in the substrate to the particle embedding film. This transfer depends crucially on the interface properties and is also limited by the onset of plastic deformation. Previous computer simulations of Co nanoparticles deposited on a Cu substrate [23] showed that strain transfer from substrate to particles depends on the structure of the interface, and that the strain is also limited to the part of the nanoparticle adjacent to the interface. To achieve large and uniform elastic deformation of the nanoparticles, it is more appropriate to embed them into a continuous film. In addition, an adhesion layer of Ta has previously been used to improve the strain transfer [24]. In this study, the Fe nanoparticles were embedded in Cu films. The procedure consisted of an initial deposition of a 10 nm thick Cu base layer on the Ta substrate, followed by deposition of the nanoparticles in a second step. Finally, the particles were capped with a 20 nm thick Cu layer. This procedure was repeated twice, in order to achieve a higher magnetic signal, resulting in a total Cu film thickness of 50 nm (see Experimental section). The Cu films may also protect the Fe particles from oxidation if exposed to atmospheric conditions. The solubility of H in Cu at equilibrium is very low [25], so we did not expect any effects from H dissolution in the Cu films during the loading procedure. Another advantage of using a Cu film is that the strain in the film can be directly measured by X-ray diffraction, which would be difficult for the Fe nanoparticles alone due to the low scattering intensity. The stress in the Cu film was determined in standard Bragg–Brentano geometry, where the in-plane stress can be calculated from the measured variation of the interplanar distance of the lattice planes parallel to the film plane. For calculation of the stress, an average elastic constant of the Cu film and the embedded Fe nanoparticles should be used. However, since the volume fraction of the Fe nanoparticles was only 0.04%, the average elastic modulus of the film with the nanoparticles was practically identical to the modulus of Cu, *E*_Cu_ = 110 GPa.

**Figure 4:**
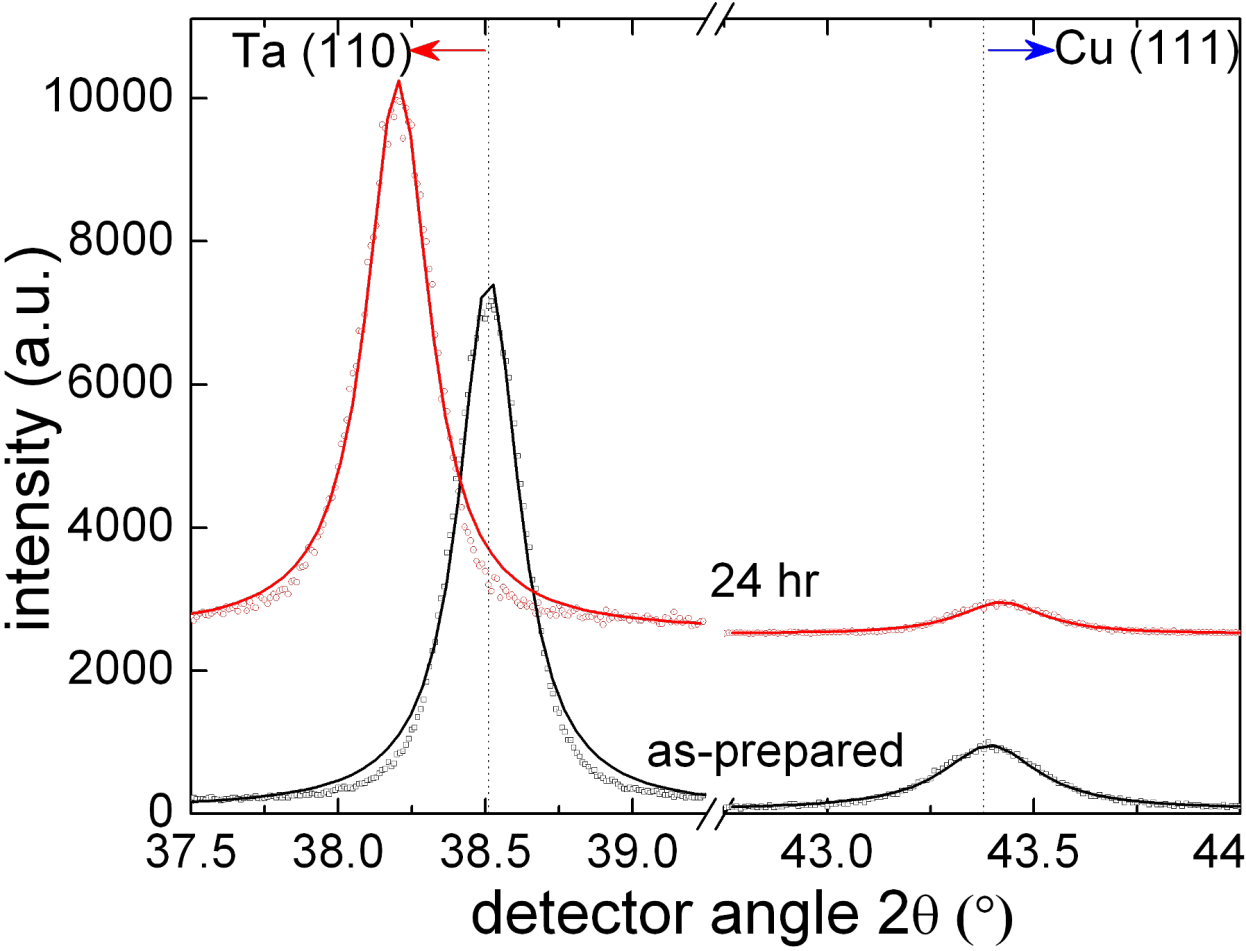
X-ray diffraction patterns (Cu Kα radiation) of Fe nanoparticles embedded in a Cu film on a Ta substrate in the as-prepared state and after 24 h of loading with hydrogen. The solid lines represent fits to the measured data using Lorentz functions.

The samples were loaded under a hydrogen pressure of 2 bar at a temperature of 100 °C for durations of up to several hours. After each loading cycle, the lattice parameters of the Ta and the Cu were determined by X-ray diffraction. The volume expansion of the Ta foils led to a shift of the Ta Bragg reflections to lower diffraction angles, while at the same time the Poisson contraction of the Cu film in the direction perpendicular to the film plane resulted in a shift of the Cu(111) fcc peak to higher angles. Both effects can be observed in Figure 4, in which X-ray diffraction patterns of the samples in the as-prepared state and after 24 h exposure to hydrogen gas are shown. The presence of a strong Cu(111) reflection indicates textured growth of the Cu layer with the close packed planes parallel to the substrate surface. The Ta(110) peak shifts from 2Θ = 38.51° to 38.20°. This corresponds to an expansion by 0.78% in the direction perpendicular to the surface. Since the Ta foil is much thicker than the Cu film, the expansion of the Ta foil occurs in the same way in the plane of the foil. The Cu film has to follow this expansion, which reflects itself in a contraction perpendicular to the film surface [21]. The observed shift of the Cu(111) peak from 2Θ = 43.383° to 43.421° corresponds to a contraction of ε_z_ = −0.083%. Applying Hooke's law for elastically isotropic media, this leads to an in-plane strain of ε_x_ = 0.09% in the Cu film. The biaxial stress can then be calculated using σ = ε_x_ *E*_Cu_/(1−ν), (where ν is Poisson’s ratio), resulting in a tensile stress value σ = +0.15 GPa for the Cu film with the embedded Fe nanoparticles. Another result is that the in-plane strain in the Cu film is lower than the strain in the Ta substrate. This can be a result of plastic deformation inside the Cu film, or of some sliding processes at the Ta/Cu interface leading to incomplete transfer of strain from the Ta to the Cu film.

### Magnetic properties

**Figure 5:**
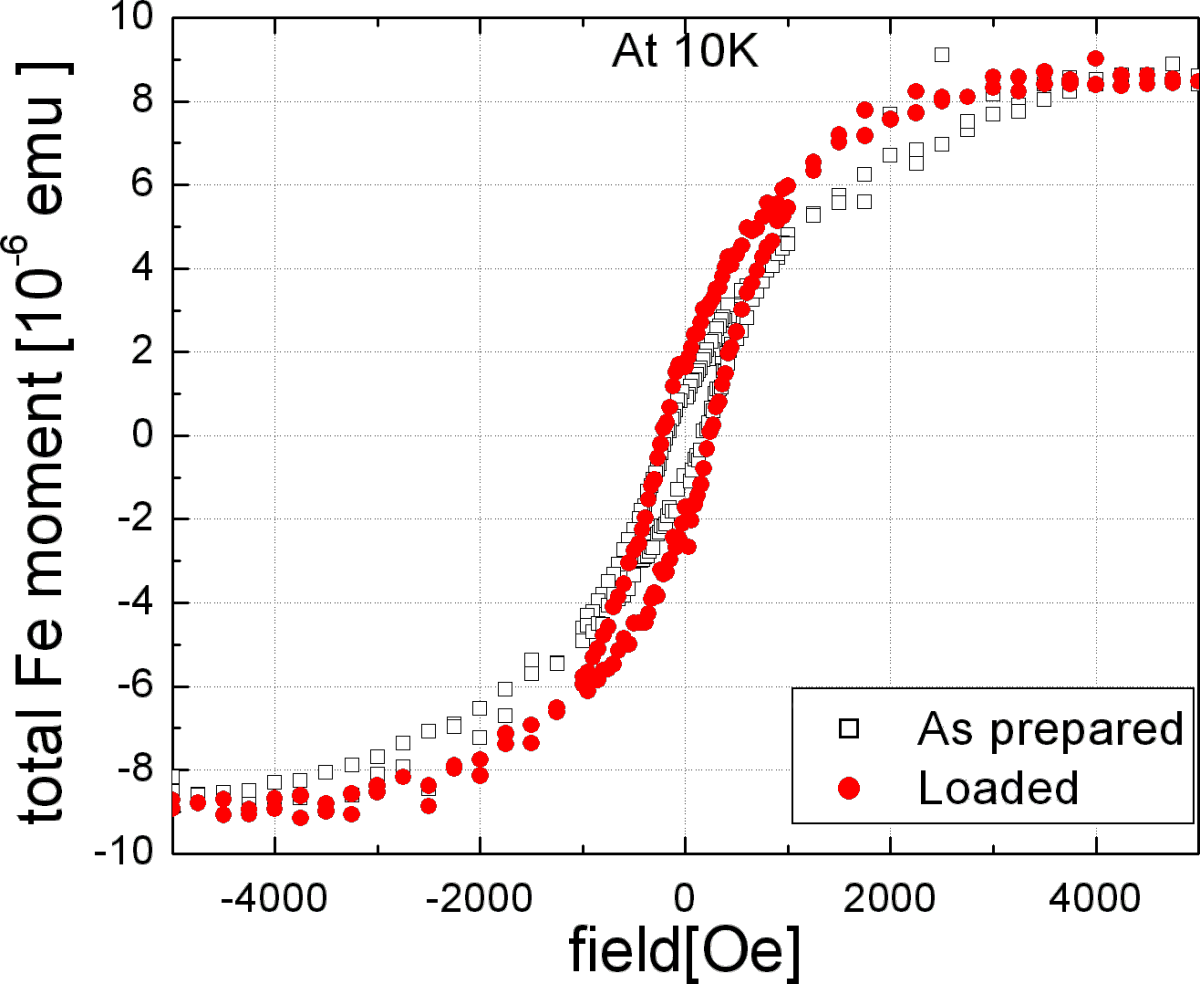
In-plane hysteresis curves of the embedded Fe nanoparticles measured at 10 K in as-prepared state (open squares) and after loading of the Ta substrate with H (red circles).

Since the H remains in the Ta substrates after loading for a long time [9], it is possible to study the effect of stress on the properties of the nanoparticle by standard ex-situ techniques after the loading process. To investigate the effect of the applied stress on the magnetic properties of the Fe nanoparticles, measurements in a SQUID magnetometer were performed over a range of temperatures. Figure 5 shows the magnetization curves of the 13 nm embedded Fe nanoparticles at a temperature *T* = 10 K; the magnetic field was applied parallel to the plane of the film (in-plane). The paramagnetic signal of the Ta substrate was fitted with a straight line and has been subtracted from the data. Note that the total ferromagnetic moment of Fe nanoparticles (8.3·10^−6^ emu) in saturation is only about 2% of the paramagnetic signal of the Ta substrate at 5000 Oe. At this temperature, the particles show a ferromagnetic behaviour. It should be noted that in the as-prepared state the Fe nanoparticles show a superparamagnetic behaviour in our SQUID measurements at 300 K. A direct comparison of the identical sample before and after hydrogen loading showed an almost identical coercivity, but a significant increase of the *M*_r_/*M*_s_ ratio (*M*_r_ is the remanence) from *M*_r_/*M*_s_ = 0.11 in the as-prepared state to *M*_r_/*M*_s_ = 0.22 in the loaded state. In addition, the saturation field *H*_s_ decreased from a value of about 4 to 5 kOe in the as-prepared state to about 2.5 to 3 kOe in the loaded state (the determination of more precise values for *H*_s_ is hindered by the scatter in the measured magnetization data). Both observations indicate a modification of the total effective magnetic anisotropy *K*_eff_ by the H loading procedure.

Considering the orientation of the applied field, it appears that the anisotropy has an additional in-plane component after H loading, which leads to a stronger tendency of the magnetization to align in the plane of the film. This can explain both the larger *M*_r_/*M*_s_ ratio (since the component of *M* parallel to the film plane is measured here) and the reduction of *H_s_*. It should be noted that the observation of these changes does not contradict the fact that the coercivity does not change. The coercivity depends on the magnetization reversal process which may, in principle, occur by rotation mechanisms restricted to the plane of the film, since the external field is applied parallel to it. In this case one would not expect to see much effect of the additional anisotropy component on the magnetization reversal process, since under the state of biaxial stress there will be no preferential orientation of the magnetization inside each particle towards any specific direction in the plane of the film. However, the fact that *H*_s_ changes is a clear indication of a modified magnetic anisotropy, since it directly measures the energy necessary for alignment of the magnetization with the applied magnetic field. From the difference between the hysteresis loops before and after the loading, the contribution of *K*_me_ can be estimated to *K*_me_ = 4·10^4^ J/m^3^, which is of similar magnitude as the effects of stress previously observed in Ni films [10]. For the calculation of *K*_me_, we have assumed here that Fe nanoparticles have the bulk saturation magnetization. We point out that the change of the shape anisotropy due to the elastic deformation of the nanoparticles does not lead to a contribution of comparable size [26].

**Figure 6:**
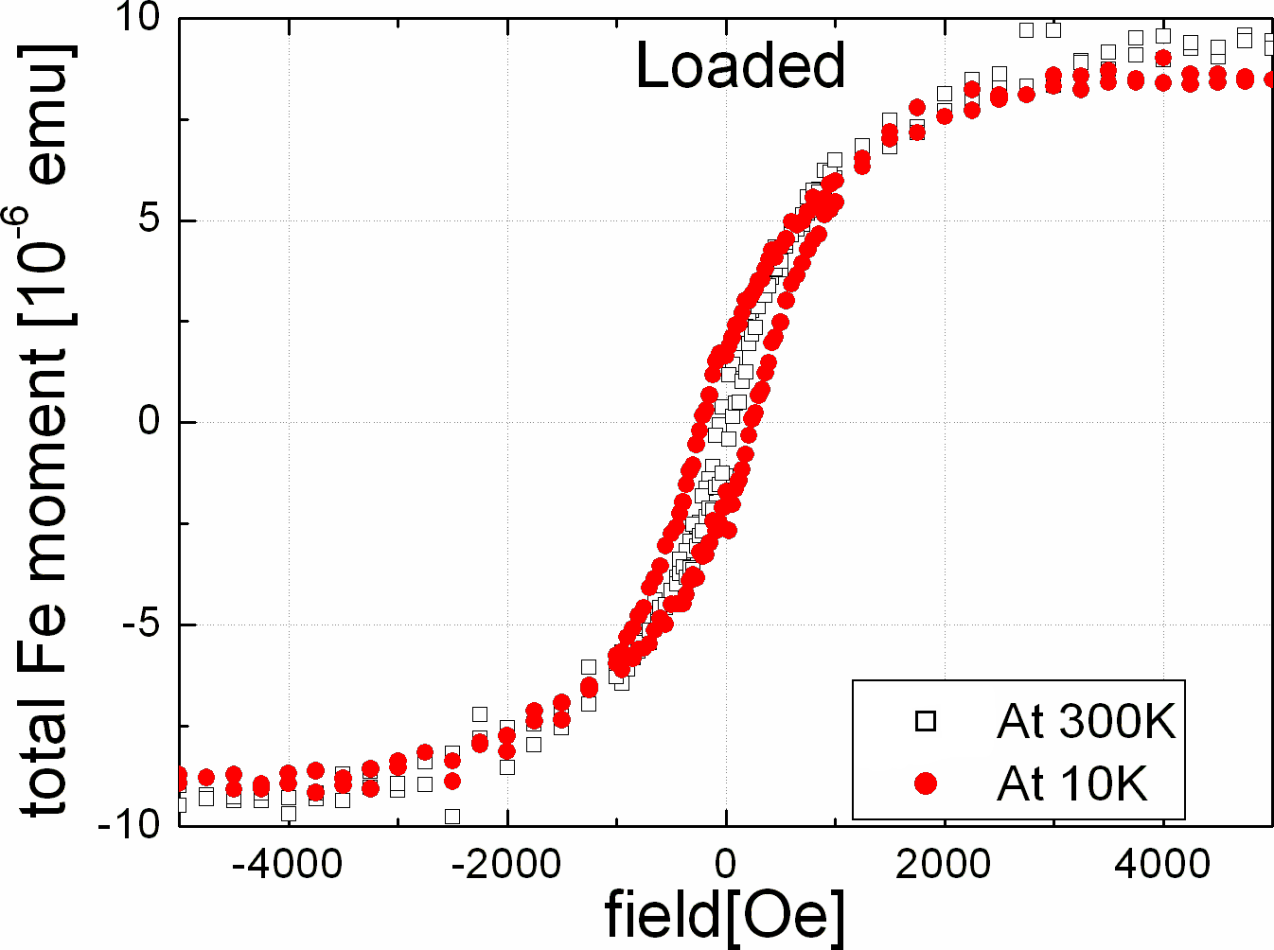
In-plane hysteresis curves of the embedded Fe nanoparticles after loading of the Ta substrate with H, measured at 10 K (filled red circles) or 300 K (open squares).

We have also studied magnetic properties of the embedded Fe nanoparticles at different temperatures. Figure 6 shows the magnetization curves for the loaded sample measured at 10 K and 300 K. Although a large reduction of the coercivity is observed at 300 K compared to 10 K, there is a small remnant magnetization indicating that at least some of the particles show ferromagnetic behaviour at 300 K. This may be attributed to the presence of a small fraction of larger Fe nanoparticles (as indicated by the size distribution given in Figure 1). We note here that there may be a small error induced by the subtraction of the large paramagnetic signal of the Ta substrate; therefore, the small difference between the magnetization curves at 300 K and 10 K for high applied fields (where the paramagnetic Ta contribution is large) is within the error of the measurement.

One characteristic feature of superparamagnetic behaviour is the existence of a blocking temperature *T*_B_, below which the magnetic fluctuations are “frozen in” on the time scale of the respective measurement. A standard technique to characterize superparamagnetic particles is the comparison of the magnetization versus temperature curves, recorded during heating from low temperature (below *T*_B_) to room temperature. The measurements are performed after previously cooling with applied field (field cooled, FC) or without an external applied magnetic field (zero field cooled, ZFC). The thermo-magnetic curves are typically measured in a small applied field. In this study, we applied a field of 100 Oe during the measurements.

**Figure 7:**
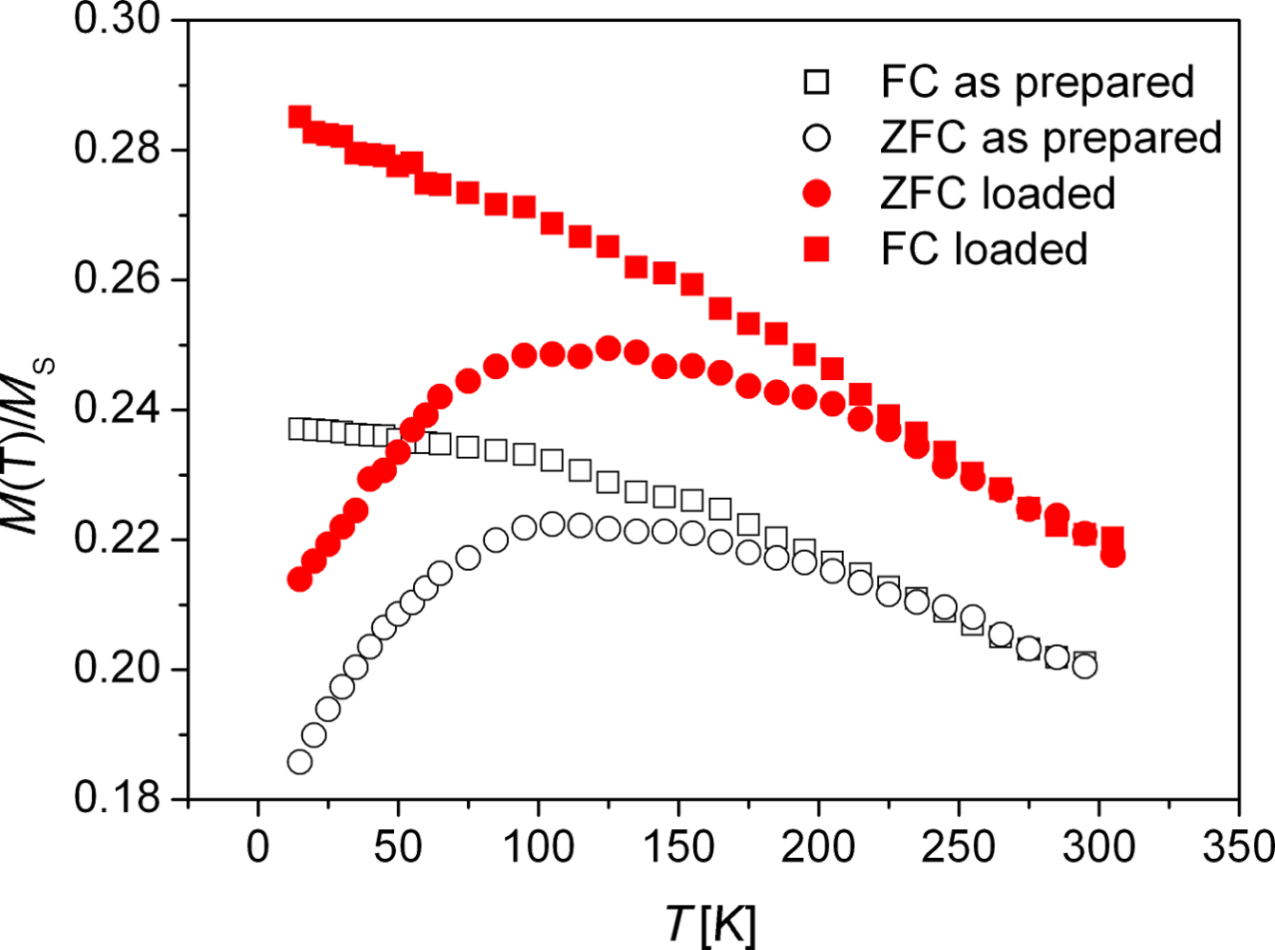
ZFC (circles) and FC (squares) magnetization curves of the Fe nanoparticles embedded in Cu film in the as-prepared state (open symbols) and after loading of the Ta substrate with H (filled red symbols) at *H* = 100 Oe. The magnetization values have been normalized to the saturation magnetization *M*_s_ at 10 K.

Figure 7 shows a comparison of the FC and ZFC curves of the same sample before and after H loading of the Ta substrate. The ZFC curves show a very similar behaviour before and after loading, whereas the FC curves differ for both samples. As a result, the temperature above which the FC and the ZFC curves fall together (called convergence temperature *T*_con_ here) shifts from about *T*_con_= 170 K in the as-prepared state to about *T*_con_ = 220 K after H loading. In fact, we do not necessarily expect to see a shift of the blocking temperature *T*_B_ (determined by the maxima of the ZFC curves) as a result of an additional magneto-elastic anisotropy contribution. As in the static magnetization measurements, we measure only the magnetization component in the direction of the applied field, which is in the plane of the film here. The fact that *T*_B_ does not change indicates that we do not “trap” the magnetization in local minima separated by a barrier. Under the applied biaxial stress, the magneto-elastic anisotropy contribution will add to the other anisotropies present, and we will only see an effect on *T*_B_ if the result would be such a “trap state”. However, since there is no preferred easy axis direction generated by the biaxial stress in the case of materials with positive magnetostriction, it is not at all evident that we should get such a state. The fact that *T*_B_ does not change indicates that the magnetization may fluctuate in the plane of the film in the same way as before the H loading. In terms of the “energy landscape” picture often used to describe the onset of superparamagnetism, this would translate to a shift of the whole landscape to a lower value without modification of the peak-to-valley differences (as far as only in-plane rotations of the magnetization are concerned). It does not, however, contradict the observed changes of *H*_s_ and the *M*_r_/*M*_s_ ratio which result from a preferred orientation of the magnetization in the plane of the film without any special easy axis direction.

If we apply the standard criterion 25 *k*_B_*T*_B_ = *K*_eff_·*V* also as a rough estimate for the effect on *T*_con_, then a shift of 50 K in *T*_con_ would correspond to an increase of the effective anisotropy energy density *K*_eff_ by approximately 1.5·10^4^ J/m^3^ for 13 nm diameter particles. This is similar to the contribution from *K*_me_ estimated from the hysteresis curves above. One may use a simple estimation of the magneto-elastic energy in the form *K*_me_ = 3/2·σ·λ, where λ is the magnetostriction constant of the material. Using λ = 8·10^−6^ as an estimate for Fe [27], the observed change of *K*_eff_ would correspond to a stress of about 1 GPa, which is larger than the average stress in the Cu film as estimated from the X-ray diffraction experiments. It is unclear at the moment whether this is due to a deviation of the local stress value in the Fe nanoparticles from the average stress in the Cu film, or due to a deviation of λ from the value for bulk Fe.

It is interesting to compare these results with earlier research on Fe nanoparticles. A recent report on the structure, morphology and magnetic properties of Fe nanoparticles deposited on single crystal surfaces can be found in [28]. In earlier studies, Methling et al. [29] observed the onset of superparamagnetism in size-selected Fe clusters at room temperature for sizes below 11 nm. However, the particles studied here, of nominally 13 nm diameter, behave superparamagnetically in the as-prepared state at room temperature. The difference may result from a slight over estimation of the average size in this study due to the method used (evaluation of SEM images). Such an over estimation could also arise from a non-spherical shape of the nanoparticles, as has been observed in [30] where a height-to-width ratio of 0.85 was found for Fe nanoparticles produced under similar conditions.

Another interesting point is the low value of the *M*_r_/*M*_s_ ratio observed for our Fe nanoparticles. According to the Stoner–Wohlfarth model, a value of *M*_r_/*M*_s_ = 0.5 would be expected for a random distribution of the easy axis for particles with uniaxial anisotropy. For the Fe nanoparticles investigated here, a cubic anisotropy is expected which would further increase the ratio of *M*_r_/*M*_s_. The low value found here could result from dipolar interactions between particles stacked vertically above each other (which might occur during the deposition of the second layer of Fe nanoparticles). Dipolar interaction can also influence the dynamic behavior of the magnetization [31]. In addition, the Fe nanoparticles may also experience Ruderman–Kittel–Kasuya–Yosida (RKKY) like coupling through the Cu matrix, which depends on the details of the arrangement of the particles. On the other hand it is well-known that the particle surfaces may lead to additional anisotropies in nanoparticles. For example, in Co nanoparticles this leads to size dependent effective anisotropies [32]. As a result, the spin structure can assume a non-collinear state as a minimum energy configuration, which would also lead to a reduction of the *M*_r_/*M*_s_ ratio. A recent study of the properties of individual Fe nanoparticles by photoemission spectroscopy [33] indicates that, depending on the size of the nanoparticles, different spin structures may result. It should be mentioned here that the presence of sub-particles with different lattice orientation in our samples may lead to a reduction of the crystalline anisotropy according to the random anisotropy model, which is commonly applied to explain the properties of nanocrystalline soft magnets [34]. This effect could further increase the influence of local surface anisotropies on the local spin structure.

Finally, it is noted that the contribution of *K*_me_ to the anisotropy in magnetic nanoparticles may be combined with any of the other contributions as given in Equation 1. It may therefore be useful for the optimization of the magnetic properties of future magnetic data storage media.

## Conclusion

In conclusion, it has been demonstrated that large biaxial stress, as a result of hydrogen loading of the substrate of embedded Fe nanoparticles in the size regime of 13 nm, leads to a modification of their magnetic properties. Results of static magnetization measurements have been presented showing large increases of the *M*_r_/*M*_s_ ratio and a reduction of the saturation field of the nanoparticles. The temperature dependent magnetization curves obtained after field cooling are also influenced by the applied stress. The results may be explained by an additional magneto-elastic anisotropy which leads to an “easy plane” rather than an “easy axis”. The results may be useful for the optimization of the magnetic properties of future magnetic data storage media.

## Experimental

Iron (Fe) nanoparticles were generated using a custom-built plasma gas condensation (PGC) chamber. Iron metal “vapour” was generated using a 2" magnetron sputter source (MAK II) which was loaded with a 99.95% pure Fe target. A continuous argon (Ar) gas stream, adjusted using a mass flow controller, was used as the sputtering gas source and also acts as a condensation gas. A constant Ar pressure of 0.85 mbar was maintained in the PGC chamber for generating Fe nanoparticles. At this Ar pressure iron metal vapour, generated at a sputtering power of 30 W, condenses in to small nuclei, which grow further by adding Fe atoms or cluster–cluster aggregation. The distance between the first aperture in the PGC chamber and the sputter source (called the aggregation length) influences the particle size. In this study, an aggregation length of 170 mm was used. Nanoparticles formed in the PGC chamber were transported to the deposition chamber by maintaining a lower pressure in the deposition chamber compared to PGC chamber. The base pressure of the deposition chamber was lower than 1·10^−7^ mbar. Using another 4" sputter source and a thermal evaporator, both located inside the deposition chamber, thin films could be deposited on the substrate simultaneously with the Fe particles. In this way, embedding of the Fe nanoparticles on the chosen substrate into a protective film could be achieved. For structural analysis in the TEM, Fe nanoparticles were deposited on copper TEM grids covered with holey carbon films, and subsequently covered by deposition of a 5 nm thick SiO*_x_* film on top using thermal evaporation. Ta foil of 200 µm thickness was used as a substrate for the stress application. One side of the Ta foil was polished to an RMS roughness of less than 5 nm using chemo-mechanical polishing. The other side of the Ta foil was coated with 100 nm of palladium, which acts as a catalyst for the hydrogen loading. Afterwards a 10 nm thick Ta adhesion layer was deposited on the polished side by DC sputtering. On top of the Ta layer, a 10 nm Cu layer was thermally evaporated. Fe nanoparticles generated in the PGC chamber were then deposited on the Cu layer for 300 s. After deposition, the Fe nanoparticles were covered with a 20 nm thick Cu layer. To get a reasonably large magnetic signal for the magnetic measurements, another set of Fe nanoparticles was deposited using the same parameters as before. Finally, a 20 nm thick Cu layer was deposited as a protective cover.
